# Normal ranges of left ventricular strain in children: a meta-analysis

**DOI:** 10.1186/s12947-015-0029-0

**Published:** 2015-08-07

**Authors:** Haki Jashari, Annika Rydberg, Pranvera Ibrahimi, Gani Bajraktari, Lindita Kryeziu, Fisnik Jashari, Michael Y. Henein

**Affiliations:** 1 Department of Public Health and Clinical Medicine, Umeå University, Umeå, Sweden; 2 Department of Clinical Sciences, Umeå University, Umeå, Sweden; 3 Department of Neonatology, Gynecology Clinic, University Clinical Center of Kosovo, Prishtina, Kosovo

**Keywords:** Strain, Echocardiography, Children, Left ventricle

## Abstract

**Aims:**

The definition of normal values of two-dimensional speckle-tracking echocardiography derived left ventricular (LV) deformation parameters, is of critical importance for the routine application of this modality in children. The objectives of this study were to perform a meta-analysis of normal ranges for longitudinal, circumferential and radial strain/strain rate values and to identify confounders that may contribute to differences in reported measures.

**Methods and Results:**

A systematic search was conducted. Studies describing normal healthy subjects and observational studies that used control groups as a comparison were included. Data were combined using a random-effect model. Effects of demographic, clinical and equipment variables were assessed through meta-regression. The search identified 1,192 subjects form 28 articles. Longitudinal strain (LS) normal mean values varied from -12.9 to -26.5 (mean, -20.5; 95% CI, -20.0 to -21.0). Normal mean values of circumferential strain (CS) varied from -10.5 to -27.0 (mean, -22.06; 95% CI, -21.5 to -22.5). Radial strain (RS) normal mean values varied from 24.9 to 62.1 (mean, 45.4; 95% CI, 43.0 to 47.8). Meta-regression showed LV end diastolic diameter as a significant determinant of variation for LS. Longitudinal systolic strain rate (LSRs) was significantly determined by the age and RS by the type of vendor used.

**Conclusion:**

Variations among different normal ranges were dependent on the vendor used, LV end-diastolic diameter and age. Vendor-independent software for analyzing myocardial deformation in children, using images from different vendors would be the ideal solution for strain measurements or else using the same system for patient’s follow up.

## Introduction

As left ventricular (LV) function is an important predictor of clinical outcome in various systemic and congenital heart diseases, new echocardiographic modalities and techniques are developed to help accurate assessment of segmental and global myocardial deformation. The most recent development is the two-dimensional speckle-tracking echocardiography (2D STE), which is a relatively angle-independent method for assessing myocardial strain and is used to quantify deformation function [1–4]. STE has been shown accurate in detecting subclinical myocardial dysfunction when most of the conventional echocardiographic parameters were normal or reported inconsistent results [5]. Likewise, detailed analysis of LV myocardial deformation in three-dimensional fashion carries the potential of providing a clearer understanding of segmental and regional function interaction in different diseases [6, 7]. However, routine application of myocardial strain in clinical practice requires clear definition of normal values and any associated variations. Of the commonest potential variables that may influence strain measurements, are patient demographics (age, gender and ethnicity) and clinical parameters (e.g. heart rate, weight, body surface area, blood pressure, LV volumes, LV dimensions and LV mass). Furthermore, technical variables i.e. vendor-customized software, probe frequency, tissue tracking and frame rate have been shown to play an important role in influencing absolute deformation measurements [8–10].

## Objectives

The objective of this study was to conduct a meta-analysis of normal ranges of LV myocardial deformation measurements (strain and strain rate) derived from 2D STE in longitudinal, circumferential and radial planes in children and to identify confounders that may contribute to differences and variability in the reported measures.

## Methods

MEDLINE, Embase, Scopus, Cochrane Central Register of Controlled Trials and ClinicalTrials.gov were searched using the key terms “strain”, “speckle tracking”, “2DSTE”, “myocardial deformation”, “echocardiography”, “left ventricle”, “children”, “infant”, “newborn”,”neonate”, “toddler” and “adolescent”. The search was completed in May 2015. No language filter was applied. Two investigators (HJ, PI) independently examined the resulted articles. To ensure accurate identification of all relevant articles, the reference lists of the articles were manually searched to further identify studies of interest that comply with the inclusion criteria.

### Study selection

Only articles reporting LV strain using 2D speckle-tracking echocardiography in normal healthy children were included. Also, this meta-analysis incorporated studies that explicitly described normal healthy subjects as well as observational studies that used control groups as a comparison for study population. Since myocardial deformation parameters were reported to change between different age groups, classifications previously used by Marcus et al. [11] and Klitisie et al. [12] (<1 year, 1-4, 5-9, 10-14 and 15-19 years) were adopted. In addition, neonates were reported as a separate age group. Healthy controls from individual studies were then allocated in respective age groups based on their mean/average age.

The commonly used 2D STE measures included were LS (Longitudinal strain) and LSRs (Longitudinal systolic strain rate). Nine studies measured global longitudinal LV strain in apical long-axis and 2- and 4-chamber views using automated function imaging, a novel 2-dimensional speckle tracking algorithm (bull’s eye). Other studies used only 4-chamber view to measure LS, which represents an average of three septal and three lateral segmental strains. Hence, in order to estimate LV longitudinal segmental strain, we averaged the values from the base, mid-cavity and apical segments of lateral and septal walls to obtain comparable patterns with the bull’s eye model.

Since, global circumferential and radial strain was reported only in 8 and 3 datasets respectively, when also considering different age groups, it seemed not eligible for conducting a Meta analysis. However, circumferential and radial strain at the level of papillary muscles could be extracted from 24 and 18 datasets respectively, hence, Meta analysis of mid-level short axis deformation parameters was conducted.

GE echocardiograph was used in 24 studies, MyLab, Siemens and Philips in one study each. One study used three different types of echocardiographs and analyzed them using Tomtec system, a vendor independent software (Table 2).

### Data collection

Clinical and echocardiographic data of interest were extracted by one investigator (HJ) and verified by a second investigator (PI). Mean LS (Longitudinal strain), LSRs (Longitudinal systolic strain rate) as well as deformation measurements at the papillary muscle level including; CS (Circumferential strain), CSRs (Circumferential systolic strain rate), RS (Radial strain) and RSRs (Radial systolic strain rate) were extracted from paper’s text or tables. When only segmental measures were available, the global longitudinal strain was calculated by pooling means and variances. A comparison between LV longitudinal segments degree of basal-to-apex gradient was conducted for all studies when available. For any unclear information, we requested additional information from the study investigators and allowed two weeks for them to respond. When they did not respond within two weeks, we used the information available.

After full text revision of preliminary selected studies further filtration was conducted.

- In the absence of corresponding author’s response, studies with incomplete data were not included [13–15] as well as only the largest study of the same author was included [16, 17].
- Studies that did not measure longitudinal strain of the whole LV were not included for LS/LSRs analysis [18, 19].
- Studies where only Global CS and/or RS were available and the strain at the level of papillary muscles could not be extracted were not included for that analysis [20, 21].
- One study was not included due to highly irrelevant data presented [22].

### Quality assessment of studies

An adopted Downs and Black score suitable for meta-analysis of normal ranges was used [23, 8] (Appendix 1). Not all included studies represented high level of quality, because of missing some important data for qualitative assessment (Appendix 2). However, all studies clearly defined the objectives, the primary outcomes and the main findings. All studies also reported patient characteristics and described the confounding factors even though heart rate, blood pressure and LV mass were only partially reported (Table 1). Standard deviation was used as an Estimate of variability of strain in all studies. Heterogeneity was not determined in any of the studies. In 11 of 28 studies sonographers were blinded to the outcome but individual blindness could not be determined in any study. Intra and inter-observer variability was conducted in 18 studies.

**Table 1:** Studies’ Characteristics

Article	Age group Study	Year	No	Age	Age group	Males %	HR	BSA/Weight	SBP	DBP	LV mass	EDV	ESV	EDD	ESD	Control studied	Disease studied
											g/m^2^	ml/m^2^	ml/m^2^				
1	Bussadori [30]	2009	15	8 ± 2 y	5-9 y	53										Healthy Subjects	Healthy Pediatric Cohort
2	Pettersen [21]	2009	22	12.7 ± 2.3 y	10-14 y	63		1.44 ± 0.24	108 ± 12	63 ± 7		58 ± 8	26 ± 4			Healthy Controls	Transposition of the Great Arteries
3	Jin Yu [19]	2010	35	2.6 ± 1.6 y	1-4 y	48		0.59 ± 0.15			59.5 ± 12.2			31.1 ± 5	19.7 ± 3.3	Healthy Controls	Kawasaki Disease
4	Van der Hulst [35]	2010	19	14.1 ± 2.4 y	10-14 y	63		1.6 ± 0.3				95 ± 10	42 ± 7			Healthy Controls	Tetralogy of Fallot
5	Koh [32]	2010	9	5.5 ± 5.5 y	5-9 y	22		0.81 ± 0.44								Healthy Controls	Left ventricular non-compaction
6	Singh [41]	2010	14	15 y	15-19 y	45	75	1.22 (1.1-1.6)								Healthy Controls	Single ventricle s/p Fontan procedure.
7	Cheung [44]	2010	44	16.4 ± 6.9 y	15-19 y	48					54.1 ± 11.9			45 ± 5	28 ± 4	Healthy Controls	Anthracycline Therapy
8	Takayasu [29]	2011	12	7.4 ± 1.7 y	5-9 y	66						46.7 ± 4.8	13.5 ± 1.5			Healthy Controls	Repaired Tetralogy of Fallot
8	Takayasu [29]	2011	19	15.5 ± 4.1 y	15-19 y	70						54.9 ± 7.7	15 ± 2.6			Healthy Controls	Repaired Tetralogy of Fallot
9	Marcus [11]	2011	24	0.3 ± 0.3 y	0-1 y	54	118	0.32 ± 0.1	82.8 ± 8	56 ± 6	36.2 ± 12.1					Healthy Subjects	Healthy Pediatric Cohort
9	Marcus [11]	2011	34	2.9 ± 1.0 y	1-4 y	56	101	0.62 ± 0.1	98 ± 10	62 ± 10	48.5 ± 11.6					Healthy Subjects	Healthy Pediatric Cohort
9	Marcus [11]	2011	36	7.2 ± 1.2 y	5-9 y	69	84	0.93 ± 0.12	104 ± 8	70 ± 8	57.2 ± 12.3					Healthy Subjects	Healthy Pediatric Cohort
9	Marcus [11]	2011	29	12.8 ± 1.6 y	10-14 y	55	77	1.43 ± 0.23	110 ± 10	72 ± 8	59.9 ± 14.2					Healthy Subjects	Healthy Pediatric Cohort
9	Marcus [11]	2011	21	17 ± 1.3 y	15-19 y	43	65	1.81 ± 0.2	116 ± 10	75 ± 9	71.8 ± 16					Healthy Subjects	Healthy Pediatric Cohort
10	Di Salvo [34]	2012	45	11 ± 3 y	10-14 y	65	78	1.32 ± 0.4	107 ± 14	61 ± 8	72 ± 15			38.9 ± 6.2		Healthy Controls	Heterozygous Familial Hypercholesterolemia
11	Fernandes [45]	2012	71	10 ± 5 y	10-14 y											Healthy Controls	Tetralogy of Fallot
12	Poterucha [37]	2012	19	15.3 ± 3 y	15-19 y	57	62	1.7 ± 0.3	119 ± 12					48 ± 5	30 ± 3.4	Healthy Controls	Anthracycline Chemotherapy
13	Hirth [43]	2012	34	11.7 ± 3.8 y	10-14 y	62	70	1.41 ± 0.39			72.3 ± 16.9					Healthy Controls	Renal Transplantation in Childhood
14	Schubert [27]	2013	30	135-207 h	Neonates	36										Healthy Neonates	Fetuses
15	Sehgal [17]	2013	21	2-5 d	Neonates			3912 g								Healthy Controls	Asphyxiated Infants
16	Klitsie [28]	2013	28	1-3 d	Neonates	36	123	3500 g								Healthy Subjects	Healthy Newborns
17	Klitsie [12]	2013	37	0.1-0.3 y	0-1 y	46	148	0.3 ± 0.1								Healthy Subjects	Healthy Pediatric Cohort
17	Klitsie [12]	2013	35	2.3-3.8 y	1-4 y	54	107	0.6 ± 0.1								Healthy Subjects	Healthy Pediatric Cohort
17	Klitsie [12]	2013	37	6.2-8.2 y	5-9 y	54	88	0.9 ± 0.2								Healthy Subjects	Healthy Pediatric Cohort
17	Klitsie [12]	2013	45	11.1-13.8 y	10-14 y	76	71	1.4 ± 02								Healthy Subjects	Healthy Pediatric Cohort
17	Klitsie [12]	2013	18	15.9-16.9 y	15-19 y	44	71	1.8 ± 0.2								Healthy Subjects	Healthy Pediatric Cohort
18	Singh [42]	2013	20	12.9 y	10-14 y	45	75	1.22	109 ± 11	68 ± 5						Healthy Subjects	Tricuspid Atresia s/p Fontan procedure
19	Van der Ende [5]	2013	40	8.4 ± 4 y	5-9 y	53		1.07 ± 0.34								Healthy Controls	Aortic Stenosis and Coarctation of Aorta
20	Dogan [31]	2013	52	6.4 ± 3.8 y	5-9 y	75	101	0.87 ± 0.32			56.1 ± 18.4			33.8 ± 7.4	17.8 ± 5.8	Healthy Controls	Aortic Stenosis
21	Barbosa [36]	2013	46	11.5 ± 3.1 y	10-14 y		74		90.5 ± 10	58.1 ± 6.3				40.7 ± 5.6	25.9 ± 3.3	Healthy Controls	Obese Patients
22	McCandless [40]	2013	22	12-29 m	1-4 y	55		0.48								Healthy Controls	Kawasaki Disease
23	Ryan [46]	2013	61	5.2 ± 0.2 y	5-9 y	100	87		104 ± 1	60.9 ± 0.9						Healthy Controls	Duchenne Muscular Dystrophy
24	Simsek [38]	2013	20	16.4 ± 1.8 y	15-19 y	100	65		115 ± 10	70.5 ± 6	88 ± 14			46.7 ± 4.7	29.3 ± 4.3	Healthy Controls	Young Elite Athletes
25	Vitarelli [20]	2014	40	11.3 ± 2.8 y	10-14 y	55	76		103 ± 1.6	67.3 ± 1.4	54.1 ± 14.1	57.2 ± 9.5		41.2 ± 5.3		Healthy Controls	Hypercholesterolemic and Obese Children
26	Forsey [33]	2014	28	9.8 ± 4.4 y	5-9 y	82		1.16 ± 0.4			64.3 ± 11.5			41.8 ± 5.7		Healthy Controls	Hypertrophic Cardiomyopathy Mutations
27	Labombarda [39]	2014	79	11.8 ± 3.2 y	10-14 y	53	76	1.27 ± 0.29	109 ± 11	62.9 ± 8.1				42.5 ± 6.7		Healthy Controls	Diabetic Children
28	Binnetoglu [16]	2015	31	15 (11-16) y	15-19 y	67		1.48 ± 0.24	110 (80-129)	60 (48-80)	67.7 ± 13			42.2 ± 2.48	26.1 ± 3.74	Healthy Controls	Obese Children and Adolescents

*HR* heart rate, *BSA* Body surface area, *SBP* systolic blood pressure, *DBP* diastolic blood pressure, *EDV* end diastolic volume, *ESV* end systolic volume, *EDD* end diastolic diameter, *ESD* end systolic diameter

### Statistical analysis

The means and 95 % confidence intervals (CI) of LS, LSRs, CS, CSRs, RS, RSRs were calculated using random-effects models weighted by inverse variance [24]. Between studies heterogeneity was assessed using Cochran’s Q test, and inconsistency was measured by I^2^ which is the percentage of total variance across studies attributable to heterogeneity rather than chance [25]. The influence of different variables such as age, sex, weight (neonates), BSA (body surface area), heart rate, blood pressure, LV mass, LV diameters, LV volumes, frame rate, tissue tracking and vendor on the variation of normal strain measurements was assessed through meta regression, if they were available in at least ten studies.

Statistical analysis was performed using standard software packages (Comprehensive Meta Analysis version 3 software; Biostat inc., Englewood, NJ, USA), with two-tailed P values <0.05 considered significant. Analysis is presented in forest plots, which is the standard way for illustrating the results of individual studies and meta-analyses. The forest plot was used as a graphical display of the relative strengths of the effect estimates and CIs for each of the individual studies, age groups and the entire meta-analysis [26, 8]. Publication bias was assessed using funnel plots and Egger’s test.

## Results

**Fig. 1:**
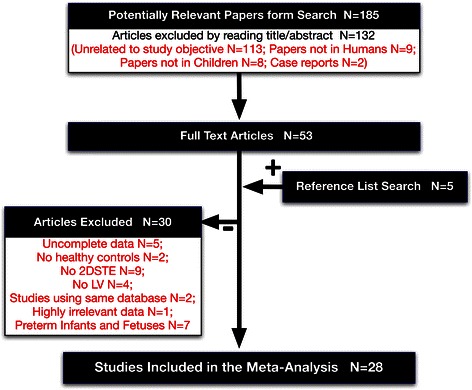
Paper selection flowchart

Our search identified 282 articles. After excluding duplicates and triplicates (n = 97), 185 studies were screened for relevance, from which only 28 fulfilled the inclusion criteria (Fig. 1). All included articles were in English language, even though no language restriction was applied. Some articles had more than one dataset of different age groups. In total, 34 datasets with 1023 subjects were eligible for meta-analysis of LS [27, 17, 28, 12, 11, 29, 30, 5, 31–35, 20, 21, 36–40, 16, 41–44] and 12 articles (13 age group studies) with 323 subjects were eligible for meta-analysis of LSRs [17, 27, 29, 30, 16, 31, 32, 21, 38, 40, 44, 43]. CS was calculated from 16 articles (24 age group studies) [28, 12, 11, 19, 31, 30, 32–34, 45, 41, 40, 46, 16, 39, 44] with a total number of 856 subjects and CSRs was calculated from six articles (six age group studies) with 233 subjects [44, 19, 45, 31, 32, 40]. 10 articles (18 age group studies) with 701 subjects were eligible for meta-analysis of RS [28, 11, 19, 31, 34, 45, 16, 44, 39, 12], while RSRs was not calculated because only three studies of different age groups contained the eligible data for meta-analysis (Tables 1 and 2).

**Table 2:** Echocardiographic characteristics

Study	Year	LV measurements	LV model for LS generation	Segmental LS	Echocardiographic View L/C, R	Vendor	Probe (Mhz)	Software	FR	Tissue tracking
Bussadori [30]	2009	L, C (S, SR)	6 segments	Yes	Apical 4CH/BMA sax	MyLab 50	2.5–3.5	Xstrain	40-64	Endocardial
Pettersen [21]	2009	L, C (S, SR)	Bull’s eye	No	Apical 2,3,4 CH/BMA (G) sax	GE Vivid 7	NS	EchoPac	69-112	Endomyocardial
Jin Yu [19]	2010	C, R (S, SR)	N/A	N/A	/Basal and Mid-level sax	GE Vivid 7	5 and 7	EchoPac	40-100	NS
Singh [41]	2010	L, C, (S)	Bull’s eye	Yes	Apical 2,3,4CH/BMA sax	GE Vivid 7	4	EchoPac	60-90	Endomyocardial
Van der Hulst [35]	2010	L (S)	Bull’s eye	Yes	Apical 2,3,4 CH	GE Vivid 7	3.5	EchoPac	>40	Endomyocardial
Koh [32]	2010	L, C (S, SR)	6 segments	No	Apical 4CH/BMA sax	GE Vivid 7	NS	EchoPac	NS	NS
Cheung [44]	2010	L, C, R (S, SR)	6 segments	No	Apical 4CH/Mid-level sax	GE Vivid 7	NS	EchoPac	NS	NS
Marcus [11]	2011	L, C, R (S)	6 segments	Yes	Apical 4CH/Basal and Mid-level sax	GE Vivid 7	3 or 5	EchoPac	70-90	Endomyocardial
Takayasu [29]	2011	L (S, SR)	6 segments	No	Apical 4 CH	GE Vivid 7	UTD	EchoPac	NS	Endomyocardial
Di salvo [34]	2012	L, C, R (S)	6 segments	Yes	Apical 4CH/BMA sax	GE Vivid 7	1.5-4	EchoPac	75 (16)	Endomyocardial
Fernandes [45]	2012	C, R (S, SR)	N/A	N/A	/BMA sax	GE Vivid 7	NS	EchoPac	60-90	Endomyocardial
Poterucha [37]	2012	L (S)	Bull’s eye	Yes	Apical 2,3,4 CH	GE Vivid 7	NS	EchoPac	>80	NS
Hirth [43]	2012	L (S, SR)	6 segments	Yes	Apical 4CH	GE Vivid 7	UTD	EchoPac	50	NS
Schubert [27]	2013	L (S, SR)	6 segments	Yes	Apical 4CH	GE Vivid 7	4–10.5	EchoPac	176-200	Endomyocardial
Sehgal [17]	2013	L (S, SR)	6 segments	Yes	Apical 4CH	GE Vivid 7	10	EchoPac	>80	NS
Klitsie [28]	2013	L, C, R (S)	6 segments	No	Apical 4CH/Mid-level sax	GE Vivid 7	NS	EchoPac	NS	Endomyocardial
Klitsie [12]	2013	L, C, R (S)	6 segments	No	Apical 4CH/Mid-level sax	GE Vivid 7	NS	EchoPac	60-80	Endomyocardial
Singh [42]	2013	L (S)	Bull’s eye	No	Apical 2,3,4CH	GE Vivid 7	1.5-4	EchoPac	NS	NS
Van der Ende [5]	2013	L (S)	Bull’s eye	Yes	Apical 2,3,4CH	GE Vivid 7	NS	EchoPac	>45	NS
Dogan [31]	2013	L, C, R (S, SR)	6 segments	Yes	Apical 4 CH/Mid-level sax	GE Vivid 7	3.0 and 7.5	EchoPac	>60	Endomyocardial
Barbosa [36]	2013	L (S)	Bull’s eye	No	Apical 2,3,4 CH	GE Vivid 7	NS	EchoPac	44	NS
Ryan [46]	2013	C (S)	N/A	N/A	/Mid-level sax	Mix	NS	Tomtec	75	Endomyocardial
McCandless [40]	2013	L, C (S, SR)	6 segments	Yes	Apical 4CH/Mid-level sax	Siemens	NS	Syngo	NS	Endocardial
Simsek [38]	2013	L (S, SR)	Bull’s eye	No	Apical 2,3,4 CH	GE Vivid 7	2.5	EchoPac	60-100	NS
Vitarelli [20]	2014	L, C, R (S)	Bull’s eye	No	Apical 2,3,4 CH/BMA (G) sax	GE Vivid E9	1.4-4.6	EchoPac	>50	NS
Forsey [33]	2014	L, C (S)	6 segments	Yes	Apical 4CH/BMA sax	GE Vivid 7	NS	EchoPac	33-129	Endomyocardial
Labombarda [39]	2014	L, C, R (S)	6 segments	No	Apical 2,3,4CH/Mid-level sax	Philips iE33	1-5	QLab	NS	Endo-Epicardial
Binnetoglu [16]	2015	L, C, R (S)	6 segments	Yes	Apical 4 CH/Mid-level sax	GE Vivid 7	NS	EchoPac	60-90	NS

*L* Longitudinal, *C* Circumferential, *R* Radial, *S* Strain, *SR* Strain rate, *CH* Chamber view, *FR* Frame rate, *BMA* Basal, medium and apical level, *BMA* (*G*) only global strain value available, *Mix* three different vendors used (GE, Philips and Acuson), *sax* short axis, *NS* not specified, *UTD* unable to determine

### Normal ranges

#### LV longitudinal myocardial deformation

**Fig. 2:**
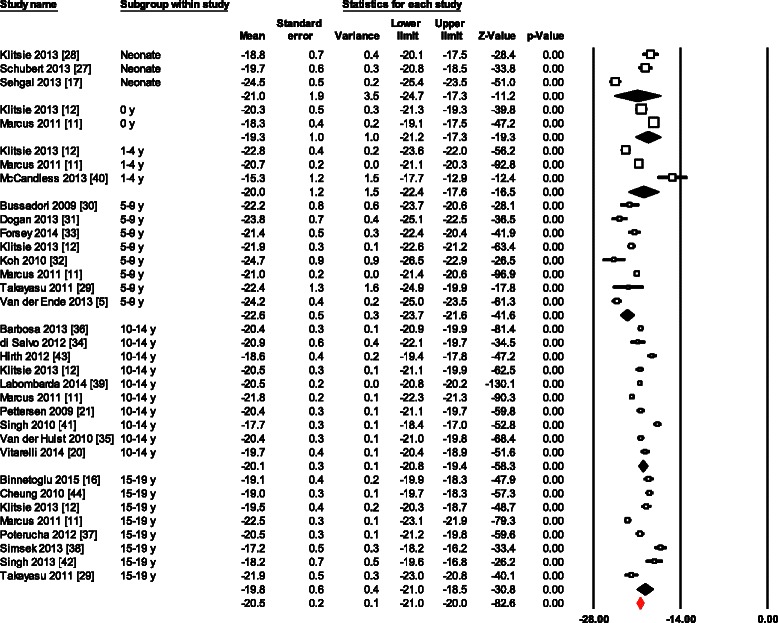
Normal value of LS. The forest plot lists the names of the studies in subgroups. The means and CIs including the results for variance, used in the inverse variance correction are shown

**Fig. 3:**
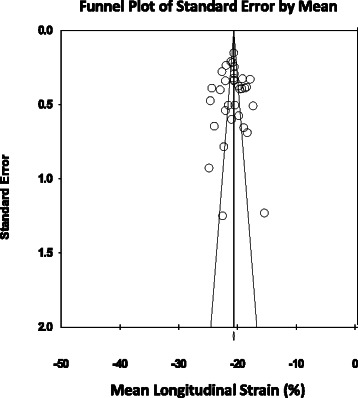
Funnel plot for studies of LS

Normal mean values of LS for all 34 age group studies combined varied from -12.9 to -26.5 (mean, -20.5; 95 % CI, -20.0 to -21.0). Overall study heterogeneity (within and between age group studies) as shown by Cochran’s Q test was 596 (*p* < 0.0001) and inconsistency by I^2^ was 94.47 % (Fig. 2). The meta-regression analysis showed that the LVEDD (left ventricular end-diastolic diameter) (β = 0.3; 95 % CI, 0.13 to 0.47; *p* < 0.001) and probe frequency (β = -0.7; 95 % CI, -1.29 to -0.1; *p* = 0.02) were the only significant determinants of variations between reported ranges of LS measurements (Table 3). There was no evidence for publication bias (*p* = 0.88) as shown by the Egger’s test for the LS (Fig. 3).

**Table 3:** Meta-regression results (p values). Significant p-values are bolded for emphasis

	LS		LSRs		CS		RS	
Variable	b (95 % CI)	p	b (95 % CI)	p	b (95 % CI)	p	b (95 % CI)	p
Age	0.06 (-0.03 to 0.16)	0.18	0.04 (0.02 to 0.05)	**<0.001**	-0.07 (-0.26 to -0.11)	0.45	0.16 (-1.01 to 1.33)	0**.**78
Male gender	0.02 (-0.02 to 0.06)	0.31	0.002 (-0.004 to 0.009)	0.49	-0.01 (-0.07 to 0.05)	0.76	0.13 (-0.7 to 0.95)	0.75
BSA	-0.24 (-1.64 to 1.15)	0.73			-1.71 (-4.25 to 0.82)	0.18	0.14 (-15.59 to 15.87)	0.98
HR	-0.004 (-0.03 to 0.02)	0.76			0.01 (-0.02 to 0.06)	0.48	-0.12 (-0.46 to 0.21)	0.47
SBP	-0.02 (-0.08 to 0.04)	0.51						
DBP	-0.08 (-0.18 to 0.02)	0.13						
LVEDD	0.3 (0.13 to 0.47)	**<0.001**						
LV mass	0.01 (-0.05 to 0.08)	0.57			-0.03 (-0.22 to 0.15)	0.71		
LV model	0.99 (-0.18 to 2.17)	0.1	0.32 (-0.1 to 0.75)	0.14				
Tissue tracking	-0.68 (-1.79 to 0.42)	0.22						
Vendor	0.92 (-0.07 to 1.92)	0.08	0.15 (-0.1 to 0.41)	0.25	0.59 (-0.36 to 1.56)	0.22	-6.59 (-11.3 to -1.91)	**0.005**
Probe	-0.70 (-1.29 to -0.1)	**0.02**			1.36 (-0.08 to 2.81)	0.07		
FR	0.007 (-0.01 to 0.03)	0.51	-0.001 (-0.006 to 0.002)	0.40	0.07 (-0.09 to 0.23)	0.38	0.55 (-0.71 to 1.82)	0.39

**Fig. 4:**
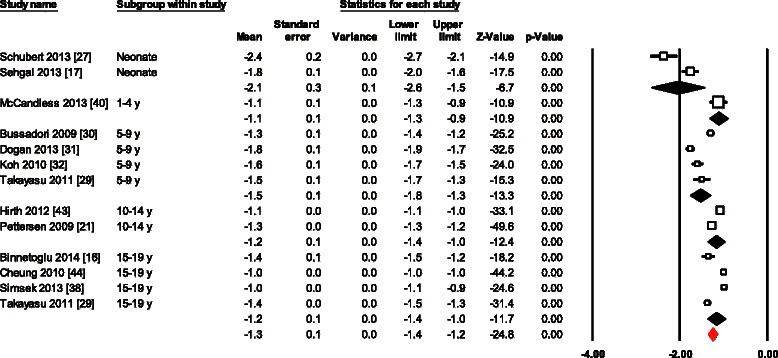
Normal value of LSRs. The forest plot lists the names of the studies in subgroups. The means and CIs including the results for variance, used in the inverse variance correction are shown

Normal values for LSRs for all 13 data sets combined varied from -0.9 to -2.7 (Mean, -1.3; 95 % CI, -1.2 to -1.4) (Fig. 4). Overall heterogeneity was 387 (*p* < 0.0001) and inconsistency was 96.9 %. Meta-regression of variables available in more than 10 studies, showed age as the only significant determinant of LSRs variations (β = 0.04; 95 % CI 0.02 to 0.05; *p* < 0.001). In addition, Egger’s test for LSRs suggested significant publication bias (p = 0.011). However, even after using Duval and Tweedie’s trim and fill, and adjusting the effect size for funnel plot asymmetry the adjusted effect was the same to the original effect.

#### LV circumferential myocardial deformation

**Fig. 5:**
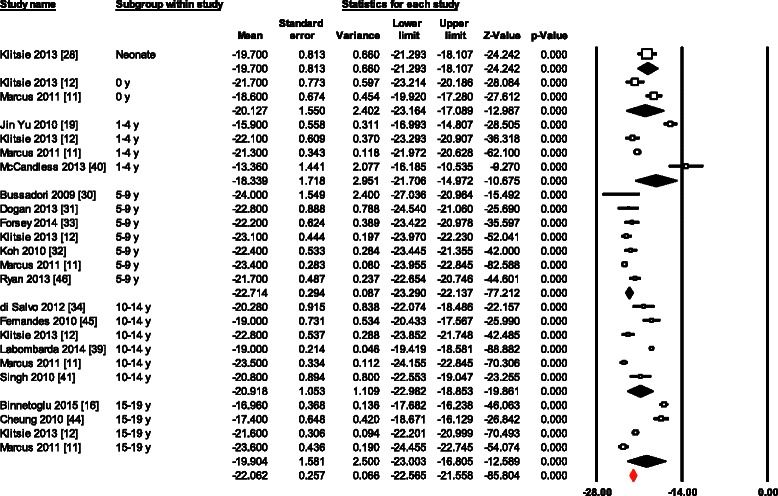
Normal value of CS. The forest plot lists the names of the studies in subgroups. The means and CIs including the results for variance, used in the inverse variance correction are shown

Normal mean values of CS for all 24 age group studies combined varied from -10.5 to -27.0 (mean, -22.06; 95 % CI, -21.5 to -22.5) (Fig. 5). Overall study heterogeneity was 599 (*p* < 0.0001) and inconsistency was 96.1 %. Meta-regression analysis did not find any significant determinant of variation among variables present in more than ten studies. There was no evidence for publication bias (*p* = 0.85).

**Fig. 6:**
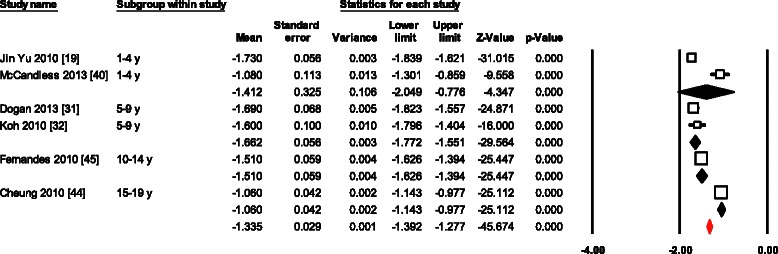
Normal value of CSRs. The forest plot lists the names of the studies in subgroups. The means and CIs including the results for variance, used in the inverse variance correction are shown

CSRs meta-analysis from six datasets of different age groups varied from -0.84 to -1.84 (mean, -1.33; 95 % CI, -1.27 to -1.39) (Fig. 6). Cochran Q test showed an overall study heterogeneity of 133 (*p* < 0.001) and I^2^ showed inconsistency of 96.25 %. Meta-regression analysis was not conducted due to small number of available studies (less than ten). There was no evidence for publication bias (*p* = 0.52).

#### LV radial myocardial deformation

**Fig. 7:**
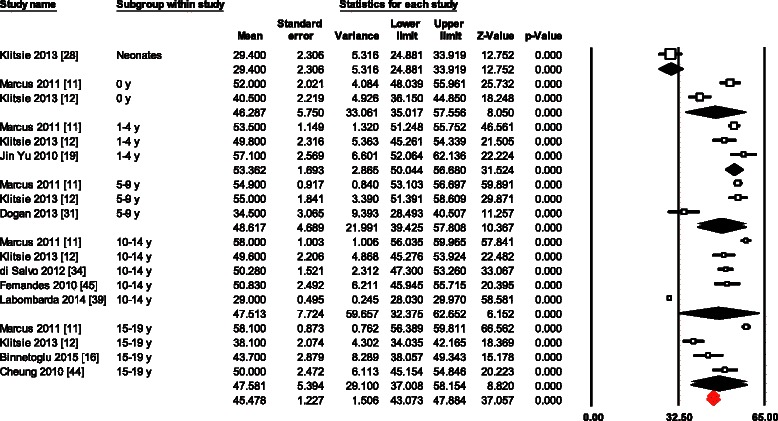
Normal value of RS. The forest plot lists the names of the studies in subgroups. The means and CIs including the results for variance, used in the inverse variance correction are shown

RS meta-analysis from 18 datasets of different age groups varied from 24.9 to 62.1 (mean, 45.4; 95 % CI, 43.0 to 47.8) (Fig. 7). Overall study heterogeneity was 1764 (*p* < 0.0001) and inconsistency was 99.03 %. Vendor was the only significant determinant of inter-study variability (*p* = 0.005). There was no evidence for publication bias (*p* = 0.122). Three studies of different age groups were not enough for meta-analysis of RSRs.

Even though all myocardial deformation parameters seemed to differ between age groups, only LSRs was significantly affected by the age of children with β = 0.04 (CI 0.02 to 0.05; *p* < 0.001). In addition, only RS resulted to be significantly influenced by the change of vendor (*p* = 0.005).

Of the LV volumes and dimensions, the LVEDD effect on LS was the only variable tested, because others were scarcely reported. LS decreased as the LVEDD increased. Higher probe frequencies were significantly related to higher LS values (*p* = 0.02). However, it turned insignificant in a bivariate model together with age (*p* = 0.07). Furthermore, examination of the LS forest plot also suggested higher variability in the neonates and infants. Low number of studies in these age groups might also play role for such variability.

There was no difference between longitudinal strain values generated by two different models, global longitudinal strain (bull’s eye model) and six-segment longitudinal strain model (-19.9 ± 0.6 vs. -20.9 ± 0.3; *p* = 0.1). Moreover, variables such as BSA, HR, SBP, DBP, LV mass, tissue tracking and FR did not significantly affected tested parameters.

### Regional longitudinal strain

Segmental strain values were available in 18 datasets. Of these, four used bull’s eye model and 14 used six-segment model. Even though, an increasing longitudinal strain from base towards apex is noticed, it was not significant for all group ages together (–19.8 % vs. -20.27 % vs. -21.27 %; *p* = 0.15). Also, in the meta-regression analysis we found that age did not significantly affect the base-to-apex gradient (*p* = 0.53). However, when analyzing LS in individual age groups, significant base-to-apex gradient was evident in 5-9 and 10-14 years age groups (*p* < 0.0001 and *p* = 0.003, respectively).

The type of vendor and tissue tracking used were just about to significantly affect the base-to-apex gradient (*p* = 0.07 and *p* = 0.06). However, since all vendors other than GE reporting on base-to-apex gradient used endocardial in spite of endomyocardial tissue tracking, such influence could not be determined if it is vendor or tissue tracking dependent. In addition, BSA, HR, SBP, DBP, LV mass, probe frequency, model used to generate LV strain and FR did not significantly affected base-to-apex gradient.

## Discussion

### Normal ranges

Deformation parameters are shown to be able to detect early subclinical dysfunction of the LV in various congenital and acquired heart diseases in children. Most of the studies have reported longitudinal strain, a very sensitive parameters of subendocardial dysfunction. In addition, evaluation of circumferential and radial strain is also important when assessing compensation patterns of LV function [6]. However, lack of a normal range of values and associated variation hinder their use for everyday clinical evaluation. This is the first study that defines LV normal strain values in children and which evaluates demographic, clinical and echocardiographic parameters as possible confounding factors through a meta-analysis. Many studies have reported normal strain values from relatively small population used as healthy control in their studies. However, combining those studies in a meta-analysis provides more reliable values of normal range than any individual study alone.

The base-to-apex LS gradient seems to emerge as children grow up. During neonatal period, there are no significant difference in longitudinal strain between LV base, mid cavity and apex. Moreover, one study has shown a decrease of base-to-apex gradient in neonates [27]. Even though, base-to-apex gradient can be noticed from early infancy, significant differences in segmental myocardial strain appear only in 5-9 and 10-14 years age groups.

### Source of bias

The meta-regression in our study showed that the vendor significantly determined the variations in RS values. In contrast, Yingchoncharoen et al. [9] in a meta-analysis of LV strain in adults refuted the role of vendor as an independent factor for differences in strain values between reports. However, earlier reports have stressed the importance of vendor variability for RS in particular [10, 47]. Therefore, since GE is the most used echocardiograph, RS normal ranges for GE vendor only are presented in Appendix 3. The higher variation in circumferential and radial strain values may be due to differences in resolution as well as spatial and temporal variation in scan-line sequencing. Smaller number of speckles (smaller area) of short axis views together with epicardial border tracking difficulties due to respiratory lung artifacts may also play a role. However, since the same degree of variability applies to all speckles, higher magnitude of variability happens to higher deformation values (radial strain) [10, 47].

Age is also another factor that influenced LSRs measures. Even though, LS, CS and RS are not significantly influenced by age, they demonstrated only a trend. Because individual studies were allocated on different age groups, the overlap of large standard deviations might explain the lack of significance when mean values were compared. We have also found large variability between neonates and infants as shown in the forest plots. This could be explained by the high sensitivity of the myocardium to changes in afterload [48]. Indeed, several hemodynamic changes occur during early neonatal life. Closure of the ductus arteriosus, with subsequent increase of pulmonary blood flow, increases preload. A postnatal afterload increase could also be related to the shifting from the low-resistance placental circulation to the increased systemic arterial blood pressure. Such dynamic changes in loading conditions are expected to influence myocardial deformation [11, 49]. In addition, lower and more variable strain values in infants could be explained on the basis of histological and physiological changes occurring early in life [50–52]. The neonatal myocardium is immature with less cellular (mitochondrial) and structural (myofibril) organization and contractile proteins [49] as well as higher Type I/Type III collagen ratio (stiffer) than adults [53]. These changes are believed to mature by 6-12 months of age, but adult values are not reached until adolescence. In addition, neonatal period is characterized by dynamic changes in multiple hormone systems [54]. Despite the small available evidence, gradually switching in energy substrate preference of the heart from fetal carbohydrates to fatty acid oxidation is another likely factor that influences cardiovascular function [55].

LS has been shown to be related to LV dimension and volume, the latter was rarely reported hence no meta-regression analysis is available. LV diastolic dimensions have been shown to inversely correlate with LS in patients with volume overload e.g. mitral regurgitation highlighting the potential need for correcting such measurements to changes in LV dimensions [56]. On the other hand, using 3D speckle tracing a positive correlation has been found between LV end systolic volumes and global CS in children [57] and between LV volume z-scores and LS [18].

Finally, the effect of heart rate, gender, body surface area, blood pressure, left ventricle mass, frame rate, tissue tracking, probe frequency and LV model of generating strain were proposed but not strongly proven. However, earlier findings [10] reported frame rate to significantly influence strain parameters, with the best reproducibility using frame rate to heart rate ratio between 0.7 and 0.9 frames/sec per beat/min [58]. The influence of blood pressure [9] and heart rate [59] on LV strain values is also reported.

## Limitations

Low number of studies to conduct meta-analysis of systolic strain rate for CS and RS is a limitation in this report, particularly early and late diastolic strain rate, which were not consistently measured. Blood pressure, LV mass, volumes, diameters and probe frequency were reported in very few studies, hence regression analysis could not be conducted for all parameters. The small number of studies using vendors other than GE might affect the regression-analysis of vendor on strain parameters because of insufficient heterogeneity. Since, STE is not user independent, the operator’s expertise might affect the values. Heterogeneity might not be seen as a limitation in meta-analysis when a pooled estimate is the main objective.

## Conclusion

In normal healthy children, the mean LS value is -20.5 (95 % CI, -20.0 to -21.0), mean LSRs is -1.3 (95 % CI, -1.2 to -1.4), mean CS is -22.06 (95 % CI, -21.54 to -22.55), mean CSRs is -1.33 (95 % CI, -1.27 to -1.39) and mean RS is 45.47 (95 % CI, 43.07 to 47.88). Variations among different normal ranges were dependent on the vendor used, LV end-diastolic dimension and age. Vendor-independent software for analyzing myocardial deformation in children, using images from different vendors would be the ideal solution for strain measurements or else using the same system for patient’s follow up.

## Appendix 1

**Table 4:** Qualitative assessment of study reporting

Description of criteria	Possible answers
Is the hypothesis/aim/objective of the study clearly described?	Yes/No
Are the main outcomes to be measured clearly described in the Introduction or Methods section?	Yes/No
Are the characteristics of the patients included in the study clearly described?	Yes/No
Are the distributions of principal confounders clearly described?	Yes/No
Are the main findings of the study clearly described?	Yes/No
Does the study provide estimates of the random variability in the data for the main outcomes?	Yes/No
Were the subjects asked to participate in the study representative of the entire population from which they were recruited?	Yes/No/UTD
Was an attempt made to blind those measuring the main outcomes of the intervention?	Yes/No/UTD
Was reproducibility analysis performed?	Yes/No

## Appendix 2

**Table 5:** Qualitative data for eligible studies

Study	Year	Objective defined?	Outcomes described?	Characteristics described?	Confounders described?	Main findings described?	Estimates of variability?	Heterogeneous population?	Sonographer(s) blinded?	Reproducibility performed?
Bussadori [30]	2009	Yes	Yes	Yes	Yes	Yes	Yes	UTD	Yes	Yes
Pettersen [21]	2009	Yes	Yes	Yes	Yes	Yes	Yes	UTD	UTD	Yes
Jin Yu [19]	2010	Yes	Yes	Yes	Yes	Yes	Yes	UTD	Yes	Yes
Singh [41]	2010	Yes	Yes	Yes	Yes	Yes	Yes	UTD	UTD	Yes
Van der Hulst [35]	2010	Yes	Yes	Yes	Yes	Yes	Yes	UTD	UTD	No
Koh [32]	2010	Yes	Yes	Yes	Yes	Yes	Yes	UTD	UTD	No
Cheung [44]	2010	Yes	Yes	Yes	Yes	Yes	Yes	UTD	UTD	No
Marcus [11]	2011	Yes	Yes	Yes	Yes	Yes	Yes	UTD	Yes	Yes
Takayasu [29]	2011	Yes	Yes	Yes	Yes	Yes	Yes	UTD	UTD	Yes
Di salvo [34]	2012	Yes	Yes	Yes	Yes	Yes	Yes	UTD	UTD	Yes
Fernandes [45]	2012	Yes	Yes	Yes	Yes	Yes	Yes	UTD	Yes	No
Poterucha [37]	2012	Yes	Yes	Yes	Yes	Yes	Yes	UTD	Yes	Yes
Hirth [43]	2012	Yes	Yes	Yes	Yes	Yes	Yes	UTD	Yes	Yes
Schubert [27]	2013	Yes	Yes	Yes	Yes	Yes	Yes	UTD	UTD	Yes
Sehgal [17]	2013	Yes	Yes	Yes	Yes	Yes	Yes	UTD	Yes	Yes
Klitsie [28]	2013	Yes	Yes	Yes	Yes	Yes	Yes	UTD	UTD	No
Klitsie [12]	2013	Yes	Yes	Yes	Yes	Yes	Yes	UTD	Yes	Yes
Singh [42]	2013	Yes	Yes	Yes	Yes	Yes	Yes	UTD	UTD	No
Van der Ende [5]	2013	Yes	Yes	Yes	Yes	Yes	Yes	UTD	UTD	Yes
Dogan [31]	2013	Yes	Yes	Yes	Yes	Yes	Yes	UTD	UTD	No
Barbosa [36]	2013	Yes	Yes	Yes	Yes	Yes	Yes	UTD	UTD	Yes
Ryan [46]	2013	Yes	Yes	Yes	Yes	Yes	Yes	UTD	Yes	Yes
McCandless [40]	2013	Yes	Yes	Yes	Yes	Yes	Yes	UTD	UTD	No
Simsek [38]	2013	Yes	Yes	Yes	Yes	Yes	Yes	UTD	Yes	Yes
Vitarelli [20]	2014	Yes	Yes	Yes	Yes	Yes	Yes	UTD	UTD	No
Forsey [33]	2014	Yes	Yes	Yes	Yes	Yes	Yes	UTD	Yes	Yes
Labombarda [39]	2014	Yes	Yes	Yes	Yes	Yes	Yes	UTD	UTD	Yes
Binnetoglu [16]	2015	Yes	Yes	Yes	Yes	Yes	Yes	UTD	UTD	No

*UTD* unable to determine

## Appendix 3

**Table 6:** Normal range of values for GE vendor

	LS (95 % CI)	LSRs (95 % CI)	CS (95 % CI)	CSRs (95 % CI)	RS (95 % CI)
Neonates	-21.0 (-17.3 to -24.7)	-2.1 (-1.5 to -2.6)	-19.7 (-18.1 to -21.3)		29.4 (24.9 to 33.9)
0-1 y	-19.3 (-17.3 to -21.2)		-20.1 (-17.1 to -23.1)		46.3 (35.0 to 57.5)
1-4 y	-21.7 (-19.7 to -23.8)		-19.8 (-16.2 to -23.3)	-1.73 (-1.62 to -1.83)	53.3 (50.0 to 56.7)
5-9 y	-22.7 (-21.5 to -23.9)	-1.6 (-1.5 to -1.8)	-22.9 (-22.5 to -23.4)	-1.66 (-1.55 to -1.77)	48.6 (39.4 to 57.8)
10-14 y	-20.0 (-19.2 to -20.9)	-1.2 (-1.0 to -1.4)	-21.3 (-19.6 to -23.1)	-1.51 (-1.39 to -1.62)	52.4 (47.4 to 57.3)
15-19 y	-19.8 (-18.5 to -21.0)	-1.2 (-1.0 to -1.4)	-19.9 (-16.8 to -23.0)	-1.06 (-0.97 to -1.14)	47.6 (37.0 to 58.1)
Overall	-20.6 (-20.1 to -21.2)	-1.4 (-1.3 to -1.5)	-22.5 (-22.0 to -22.9)	-1.42 (-1.37 to -1.47)	46.8 (44.6 to 48.9)

## Abbreviations

LV: Left ventricle/ ventricular
2D STE: Two-dimensional speckle-tracking echocardiography
2D: Two-dimensional
LS: Longitudinal strain
LSRs: Longitudinal systolic strain rate
CS: Circumferential strain
CSRs: Circumferential systolic strain rate
RS: Radial strain
RSRs: Radial systolic strain rate
CI: Confidence interval
BSA: Body surface area
FR: Frame rate
LVEDD: Left ventricular end diastolic diameter

## References

[1] Leitman M, Lysyansky P, Sidenko S, Shir V, Peleg E, Binenbaum M (2004). Two-dimensional strain-a novel software for real-time quantitative echocardiographic assessment of myocardial function. J Am Soc Echocardiogr.

[2] Reisner SA, Lysyansky P, Agmon Y, Mutlak D, Lessick J, Friedman Z (2004). Global longitudinal strain: a novel index of left ventricular systolic function. J Am Soc Echocardiogr.

[3] Marwick TH (2006). Measurement of strain and strain rate by echocardiography: ready for prime time?. J Am Coll Cardiol.

[4] Grabskaya E, Spira C, Hoffmann R, Altiok E, Ocklenburg C, Hoffmann R (2010). Myocardial rotation but not circumferential strain is transducer angle dependent: a speckle tracking echocardiography study. Echocardiography.

[5] Van der Ende J, Vazquez Antona CA, Erdmenger Orellana J, Romero Cardenas A, Roldan FJ, Vargas BJ (2013). Left ventricular longitudinal strain measured by speckle tracking as a predictor of the decrease in left ventricular deformation in children with congenital stenosis of the aorta or coarctation of the aorta. Ultrasound Med Biol.

[6] Jashari H, Rydberg A, Ibrahimi P, Bajraktari G, Henein MY (2015). Left ventricular response to pressure afterload in children: aortic stenosis and coarctation: a systematic review of the current evidence. Int J Cardiol.

[7] Leonardi B, Margossian R, Sanders SP, Chinali M, Colan SD. Ventricular mechanics in patients with aortic valve disease: longitudinal, radial, and circumferential components. Cardiology in the young. 2013:1-8. doi:10.1017/S1047951112002326. 10.1017/S1047951112002326 23388108

[8] Levy PT, Sanchez Mejia AA, Machefsky A, Fowler S, Holland MR, Singh GK (2014). Normal ranges of right ventricular systolic and diastolic strain measures in children: a systematic review and meta-analysis. J Am Soc Echocardiogr.

[9] Yingchoncharoen T, Agarwal S, Popovic ZB, Marwick TH (2013). Normal ranges of left ventricular strain: a meta-analysis. J Am Soc Echocardiogr.

[10] Koopman LP, Slorach C, Manlhiot C, McCrindle BW, Jaeggi ET, Mertens L (2011). Assessment of myocardial deformation in children using Digital Imaging and Communications in Medicine (DICOM) data and vendor independent speckle tracking software. J Am Soc Echocardiogr.

[11] Marcus KA, Mavinkurve-Groothuis AM, Barends M, van Dijk A, Feuth T, de Korte C (2011). Reference values for myocardial two-dimensional strain echocardiography in a healthy pediatric and young adult cohort. J Am Soc Echocardiogr.

[12] Klitsie LM, Roest AA, van der Hulst AE, Stijnen T, Blom NA, Ten Harkel AD (2013). Assessment of intraventricular time differences in healthy children using two-dimensional speckle-tracking echocardiography. J Am Soc Echocardiogr.

[13] Basu S, Frank LH, Fenton KE, Sable CA, Levy RJ, Berger JT (2012). Two-dimensional speckle tracking imaging detects impaired myocardial performance in children with septic shock, not recognized by conventional echocardiography. Pediatr Crit Care Med.

[14] Koopman LP, McCrindle BW, Slorach C, Chahal N, Hui W, Sarkola T (2012). Interaction between myocardial and vascular changes in obese children: a pilot study. J Am Soc Echocardiogr.

[15] Gziri MM, Hui W, Amant F, Van Calsteren K, Ottevanger N, Kapusta L (2013). Myocardial function in children after fetal chemotherapy exposure. A tissue Doppler and myocardial deformation imaging study. Eur J Pediatr.

[16] Binnetoglu FK, Yildirim S, Topaloglu N, Tekin M, Kaymaz N, Aylanc H (2015). Early detection of myocardial deformation by 2D speckle tracking echocardiography in normotensive obese children and adolescents. Anatolian J Cardiol.

[17] Sehgal A, Wong F, Menahem S (2013). Speckle tracking derived strain in infants with severe perinatal asphyxia: a comparative case control study. Cardiovasc Ultrasound.

[18] Lorch SM, Ludomirsky A, Singh GK (2008). Maturational and growth-related changes in left ventricular longitudinal strain and strain rate measured by two-dimensional speckle tracking echocardiography in healthy pediatric population. J Am Soc Echocardiogr.

[19] Yu JJ, Choi HS, Kim YB, Son JS, Kim YH, Ko JK (2010). Analyses of left ventricular myocardial deformation by speckle-tracking imaging during the acute phase of Kawasaki disease. Pediatr Cardiol.

[20] Vitarelli A, Martino F, Capotosto L, Martino E, Colantoni C, Ashurov R (2014). Early myocardial deformation changes in hypercholesterolemic and obese children and adolescents: a 2D and 3D speckle tracking echocardiography study. Medicine.

[21] Pettersen E, Fredriksen PM, Urheim S, Thaulow E, Smith HJ, Smevik B (2009). Ventricular function in patients with transposition of the great arteries operated with arterial switch. Am J Cardiol.

[22] Elkiran O, Karakurt C, Kocak G, Karadag A (2014). Tissue Doppler, strain, and strain rate measurements assessed by two-dimensional speckle-tracking echocardiography in healthy newborns and infants. Cardiol Young.

[23] Downs SH, Black N (1998). The feasibility of creating a checklist for the assessment of the methodological quality both of randomised and non-randomised studies of health care interventions. J Epidemiol Community Health.

[24] DerSimonian R, Laird N (1986). Meta-analysis in clinical trials. Control Clin Trials.

[25] Higgins JP, Thompson SG, Deeks JJ, Altman DG (2003). Measuring inconsistency in meta-analyses. BMJ.

[26] Normand SL (1999). Meta-analysis: formulating, evaluating, combining, and reporting. Stat Med.

[27] Schubert U, Muller M, Norman M, Abdul-Khaliq H (2013). Transition from fetal to neonatal life: changes in cardiac function assessed by speckle-tracking echocardiography. Early Hum Dev.

[28] Klitsie LM, Roest AA, Haak MC, Blom NA, Ten Harkel AD (2013). Longitudinal follow-up of ventricular performance in healthy neonates. Early Hum Dev.

[29] Takayasu H, Takahashi K, Takigiku K, Yasukochi S, Furukawa T, Akimoto K (2011). Left ventricular torsion and strain in patients with repaired tetralogy of Fallot assessed by speckle tracking imaging. Echocardiography.

[30] Bussadori C, Moreo A, Di Donato M, De Chiara B, Negura D, Dall'Aglio E (2009). A new 2D-based method for myocardial velocity strain and strain rate quantification in a normal adult and paediatric population: assessment of reference values. Cardiovasc Ultrasound.

[31] Dogan V, Ocal B, Orun UA, Ozgur S, Yilmaz O, Keskin M (2013). Strain and strain rate echocardiography findings in children with asymptomatic congenital aortic stenosis. Pediatr Cardiol.

[32] Koh C, Hong WJ, Wong SJ, Cheung YF (2010). Systolic-diastolic coupling of myocardial deformation of the left ventricle in children with left ventricular noncompaction. Heart Vessels.

[33] Forsey J, Benson L, Rozenblyum E, Friedberg MK, Mertens L (2014). Early changes in apical rotation in genotype positive children with hypertrophic cardiomyopathy mutations without hypertrophic changes on two-dimensional imaging. J Am Soc Echocardiogr.

[34] Di Salvo G, D'Aiello AF, Castaldi B, Fadel B, Limongelli G, D'Andrea A (2012). Early left ventricular abnormalities in children with heterozygous familial hypercholesterolemia. J Am Soc Echocardiogr.

[35] van der Hulst AE, Delgado V, Holman ER, Kroft LJ, de Roos A, Hazekamp MG (2010). Relation of left ventricular twist and global strain with right ventricular dysfunction in patients after operative “correction” of tetralogy of fallot. Am J Cardiol.

[36] Barbosa JA, Mota CC, Simoes ESAC, Nunes Mdo C, Barbosa MM (2013). Assessing pre-clinical ventricular dysfunction in obese children and adolescents: the value of speckle tracking imaging. Eur Heart J Cardiovasc Imaging.

[37] Poterucha JT, Kutty S, Lindquist RK, Li L, Eidem BW (2012). Changes in left ventricular longitudinal strain with anthracycline chemotherapy in adolescents precede subsequent decreased left ventricular ejection fraction. J Am Soc Echocardiogr.

[38] Simsek Z, Hakan Tas M, Degirmenci H, Gokhan Yazici A, Ipek E, Duman H (2013). Speckle tracking echocardiographic analysis of left ventricular systolic and diastolic functions of young elite athletes with eccentric and concentric type of cardiac remodeling. Echocardiography.

[39] Labombarda F, Leport M, Morello R, Ribault V, Kauffman D, Brouard J (2014). Longitudinal left ventricular strain impairment in type 1 diabetes children and adolescents: a 2D speckle strain imaging study. Diabetes Metab.

[40] McCandless RT, Minich LL, Wilkinson SE, McFadden ML, Tani LY, Menon SC (2013). Myocardial strain and strain rate in Kawasaki disease. Eur Heart J Cardiovasc Imaging.

[41] Singh GK, Cupps B, Pasque M, Woodard PK, Holland MR, Ludomirsky A (2010). Accuracy and reproducibility of strain by speckle tracking in pediatric subjects with normal heart and single ventricular physiology: a two-dimensional speckle-tracking echocardiography and magnetic resonance imaging correlative study. J Am Soc Echocardiogr.

[42] Singh GK, Vitola BE, Holland MR, Sekarski T, Patterson BW, Magkos F (2013). Alterations in ventricular structure and function in obese adolescents with nonalcoholic fatty liver disease. J Pediatr.

[43] Hirth A, Edwards NC, Greve G, Tangeraas T, Gerdts E, Lenes K (2012). Left ventricular function in children and adults after renal transplantation in childhood. Pediatr Nephrol.

[44] Cheung YF, Hong WJ, Chan GC, Wong SJ, Ha SY (2010). Left ventricular myocardial deformation and mechanical dyssynchrony in children with normal ventricular shortening fraction after anthracycline therapy. Heart.

[45] Fernandes FP, Manlhiot C, Roche SL, Grosse-Wortmann L, Slorach C, McCrindle BW (2012). Impaired left ventricular myocardial mechanics and their relation to pulmonary regurgitation, right ventricular enlargement and exercise capacity in asymptomatic children after repair of tetralogy of Fallot. J Am Soc Echocardiogr.

[46] Ryan TD, Taylor MD, Mazur W, Cripe LH, Pratt J, King EC (2013). Abnormal circumferential strain is present in young Duchenne muscular dystrophy patients. Pediatr Cardiol.

[47] Risum N, Ali S, Olsen NT, Jons C, Khouri MG, Lauridsen TK (2012). Variability of global left ventricular deformation analysis using vendor dependent and independent two-dimensional speckle-tracking software in adults. J Am Soc Echocardiogr.

[48] Rowland DG, Gutgesell HP (1995). Noninvasive assessment of myocardial contractility, preload, and afterload in healthy newborn infants. Am J Cardiol.

[49] Hines MH (2013). Neonatal cardiovascular physiology. Semin Pediatr Surg.

[50] Nakanishi T, Seguchi M, Takao A (1988). Development of the myocardial contractile system. Experientia.

[51] Davis PJ, Cladis FP, Motoyama EK. Smith’s Anesthesia for Infants and Children, 8th ed.87-97

[52] Nishikawa T, Sekiguchi M, Takao A, Ando M, Hiroe M, Morimoto S (1990). Histopathological assessment of endomyocardial biopsy in children: I. Semiquantitative study on the hypertrophy of cardiac myocytes. Am J Cardiovasc Pathol.

[53] Marijianowski MM, van der Loos CM, Mohrschladt MF, Becker AE (1994). The neonatal heart has a relatively high content of total collagen and type I collagen, a condition that may explain the less compliant state. J Am Coll Cardiol.

[54] Wassner AJ, Modi BP (2013). Endocrine physiology in the newborn. Semin Pediatr Surg.

[55] Onay-Besikci A (2006). Regulation of cardiac energy metabolism in newborn. Mol Cell Biochem.

[56] Marciniak A, Claus P, Sutherland GR, Marciniak M, Karu T, Baltabaeva A (2007). Changes in systolic left ventricular function in isolated mitral regurgitation. A strain rate imaging study. Eur Heart J.

[57] Zhang L, Gao J, Xie M, Yin P, Liu W, Li Y (2013). Left ventricular three-dimensional global systolic strain by real-time three-dimensional speckle-tracking in children: feasibility, reproducibility, maturational changes, and normal ranges. J Am Soc Echocardiogr.

[58] Sanchez AA, Levy PT, Sekarski TJ, Hamvas A, Holland MR, Singh GK (2014). Effects of frame rate on two-dimensional speckle tracking-derived measurements of myocardial deformation in premature infants. Echocardiography.

[59] Cantinotti M, Kutty S, Giordano R, Assanta N, Murzi B, Crocetti M (2015). Review and status report of pediatric left ventricular systolic strain and strain rate nomograms. Heart Fail Rev.

